# Fluconazole Alters the Polysaccharide Capsule of *Cryptococcus gattii* and Leads to Distinct Behaviors in Murine Cryptococcosis

**DOI:** 10.1371/journal.pone.0112669

**Published:** 2014-11-13

**Authors:** Julliana Ribeiro Alves Santos, Rodrigo Assunção Holanda, Susana Frases, Mayara Bravim, Glauber de S. Araujo, Patrícia Campi Santos, Marliete Carvalho Costa, Maira Juliana Andrade Ribeiro, Gabriella Freitas Ferreira, Ludmila Matos Baltazar, Aline Silva Miranda, Danilo Bretas Oliveira, Carolina Maria Araújo Santos, Alide Caroline Lima Fontes, Ludmila Ferreira Gouveia, Maria Aparecida Resende-Stoianoff, Jonatas Santos Abrahão, Antônio Lúcio Teixeira, Tatiane Alves Paixão, Danielle G. Souza, Daniel Assis Santos

**Affiliations:** 1 Departamento de Microbiologia, Instituto de Ciências Biológicas, Universidade Federal de Minas Gerais, Minas Gerais, Brazil; 2 Laboratório de Ultraestrutura Celular Hertha Meyer, Instituto de Biofísica Carlos Chagas Filho, Universidade Federal do Rio de Janeiro, Rio de Janeiro, Brazil; 3 Laboratório de Biotecnologia (LABIO), Instituto Nacional de Metrologia, Normalização e Qualidade Industrial (INMETRO), Rio de Janeiro, Brazil; 4 Laboratório Interdisciplinar de Investigação Médica, Faculdade de Medicina, Universidade Federal de Minas Gerais, Belo Horizonte, MG, Brazil; 5 Departamento de Patologia Geral, Instituto de Ciências Biológicas, Universidade Federal de Minas Gerais, Minas Gerais, Brazil; University of Minnesota, United States of America

## Abstract

*Cryptococcus gattii* is an emergent human pathogen. Fluconazole is commonly used for treatment of cryptococcosis, but the emergence of less susceptible strains to this azole is a global problem and also the data regarding fluconazole-resistant cryptococcosis are scarce. We evaluate the influence of fluconazole on murine cryptococcosis and whether this azole alters the polysaccharide (PS) from cryptococcal cells. L27/01 strain of *C. gattii* was cultivated in high fluconazole concentrations and developed decreased drug susceptibility. This phenotype was named L27/01**_F_**, that was less virulent than L27/01 in mice. The physical, structural and electrophoretic properties of the PS capsule of L27/01_F_ were altered by fluconazole. L27/01**_F_** presented lower antiphagocytic properties and reduced survival inside macrophages. The L27/01**_F_** did not affect the central nervous system, while the effect in brain caused by L27/01 strain began after only 12 hours. Mice infected with L27/01**_F_** presented lower production of the pro-inflammatory cytokines, with increased cellular recruitment in the lungs and severe pulmonary disease. The behavioral alterations were affected by L27/01, but no effects were detected after infection with L27/01**_F_**. Our results suggest that stress to fluconazole alters the capsule of *C. gattii* and influences the clinical manifestations of cryptococcosis.

## Introduction

Deaths due to cryptococcosis, particularly the variant caused by *Cryptococcus gattii*, an emergent fungal pathogen that affects immunocompetent individuals, are increasing [1]–[3]. This fungus is acquired from the environment through inhalation of desiccated yeast cells or spores. The lungs are the most strongly affected site, followed by the central nervous system (CNS), where meningoencephalitis is often detected [2], [4].

The therapy for cryptococcosis caused by *C. gattii* is very important for reduction of mortality [3], [5] and fluconazole is recommended for the treatment of mild to moderate pulmonary symptoms of *C. gattii*-induced cryptococcosis. Amphotericin B (alone or in combination with 5-flucytosine) followed by maintenance with fluconazole is recommended for severe cases [4] but long-term maintenance therapy with fluconazole often leads to fungal resistance [6]. Although the antifungal combination represents an important alternative to the conventional therapy [7], efficacy may also be impaired by polyenes and azoles used in combination [8]. These difficulties in treating cryptococcosis may result in treatment failure or relapse.

Fungal resistance can be confirmed *in vitro*: Resistant strains are able to grow in the presence of high concentrations of fluconazole [9], [10]. Special attention should be paid to the growing resistance to fluconazole. The ARTEMIS global antifungal surveillance study revealed increases in reduced susceptibility to fluconazole in *C. gattii* [6], but it is still unclear whether and how this phenomenon affects the disease caused by this agent.

The aim of this study was to evaluate the influence of reduced susceptibility to fluconazole on the disease caused by *C. gattii* in C57BL/6 mice and whether this azole alters polysaccharide from cryptococcal cells. Our results suggest that the development of reduced susceptibility to fluconazole alters the polysaccharide capsule and the clinical manifestations of cryptococcosis, influencing whether the disease is restricted to the lungs or is disseminated to the CNS.

## Materials and Methods

### Ethics statement

The protocol of animal studies was approved by the Comitê de Ética em Experimentação Animal (CETEA) from Universidade Federal de Minas Gerais (Protocol 170/2011) and animal experiments were performed in strict accordance with the Brazilian Federal Law 11,794 establishing procedures for the scientific use of animals. All mice were housed in clean bedding (five mice per cage) with food and water *ad libitum* in a controlled environment with a 12 h light/dark cycle at 23°C. All mice were monitored twice daily. For intratracheal inoculation, mice were anesthetized by intraperitoneal injection of ketamine hydrochloride (50 mg mL^−1^) and xylazine (0.02 mg mL^−1^) in sterile saline. All efforts were made to minimize suffering. Any mice that appeared moribund (e.g. intense piloerection, convulsions, lack of locomotor activity) were euthanized immediately. Mice were euthanized under anesthesia (i.p. injection of ketamine hydrochloride (50 mg mL^−1^) and xylazine (0.02 mg mL^−1^) in sterile saline) by cervical dislocation by experienced animal handlers.

### *Cryptococcus gattii* and antifungal drug susceptibility testing

We tested 12 strains of *C. gattii* (from the culture collection of the Laboratório de Micologia/Universidade Federal de Minas Gerais, Brazil). Initially, the MIC for fluconazole (Sigma-Aldrich, St. Louis, Missouri, USA) was determined in drug-supplemented solid culture medium in Sabouraud’s dextrose agar (SDA). The MICs for fluconazole and amphotericin B (Sigma-Aldrich) were also determined by the microdilution method [8], [11]–[13]. Drug susceptibility testing was performed in three independent experiments in duplicate.

### Reduced susceptibility to fluconazole induction

**Figure 1:**
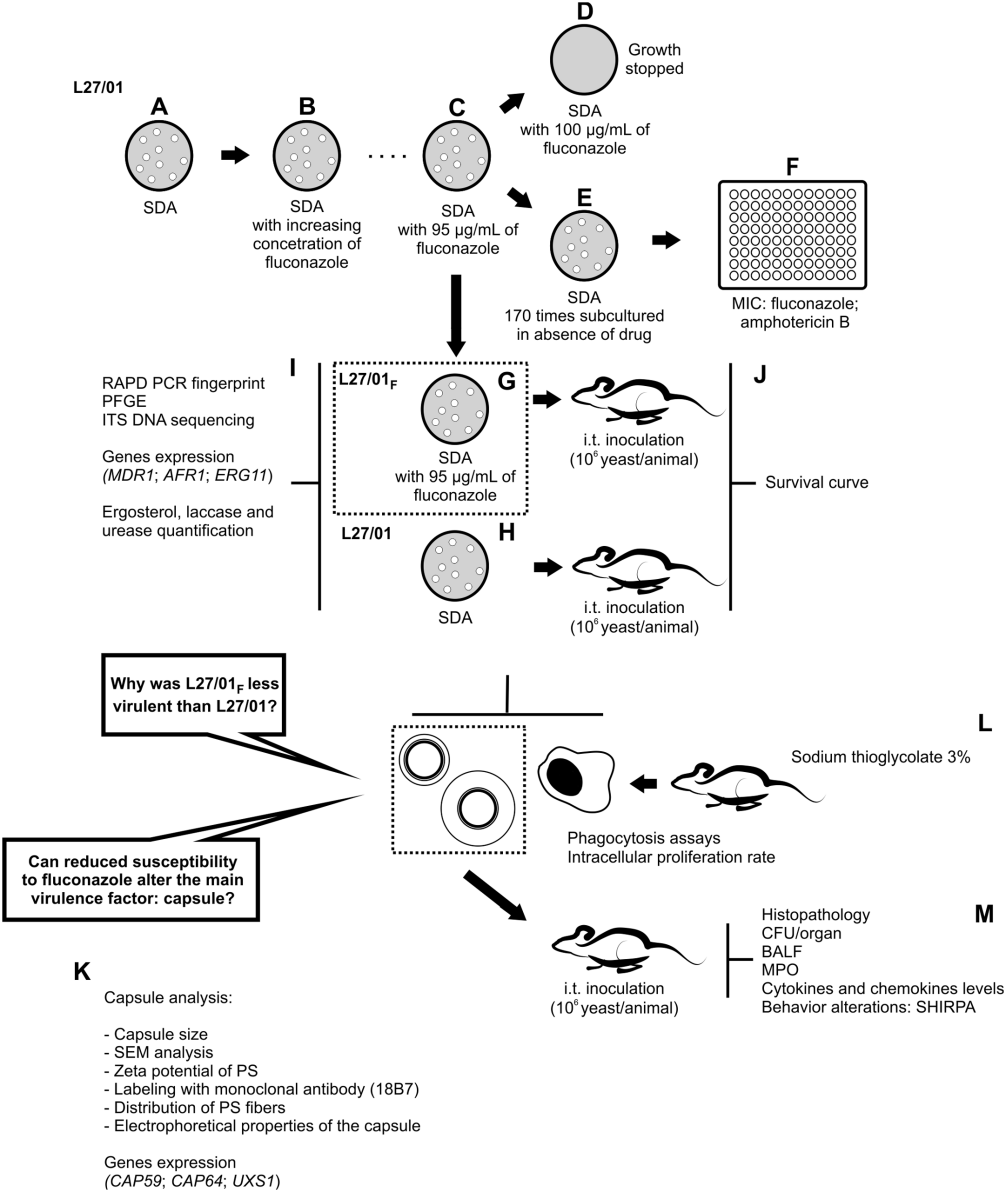
Synopsis of the methodology. Fluconazole-resistant strain selection (**A–F**). After determining the MIC of fluconazole on SDA, an average of five colonies obtained from the highest fluconazole concentration were selected for culture on SDA plates supplemented with this drug. L27/01 (**A**) was the strain able to grow at the highest fluconazole concentration (**B**) and was chosen for culture on SDA plates supplemented with this drug. This strain was cultured in solid medium with increasing concentrations (**B–C**) of fluconazole until growth ceased at 100 µg/mL (**D**). To verify the maintenance of resistance to fluconazole and cross-resistance between this drug and amphotericin B, the selected strain was cultured in SDA without drug every 48 h 170 times (**E**), and the MIC test was performed by microdilution every 5 subcultures (**F**). Colonies grown at 95 µg/mL were maintained in this concentration, and the strain was named “L27/01**_F_**” (**G**). L27/01 strain grew in the absence of drug (**H**). The genetic similarity between L27/01 and L27/01**_F_** strains was evaluated by randomly amplified polymorphic DNA (RAPD)-PCR, PFGE and ITS Sequencing. *CAP59, CAP64, ARF-1, ERG11, UXS-1* levels by real-time PCR were evaluated. Lipid evaluation was performed to compare the ergosterol content of L27/01 and L27/01**_F_** cell membranes. Also, Urease and Laccase activities of L27/01 and L27/01**_F_** strains were determined (**I**). Evaluation of murine cryptococcosis after inoculation with L27/01 or L27/01**_F_** strain: survival curve (**J**). Investigation of whether fluconazole affects the polysaccharide (PS) capsule (**K**). The phagocytosis assay was performed to assess the influence of PS capsules from L27/01 and L27/01**_F_** strains on phagocytosis and intracellular proliferation rate (IPR) in murine peritoneal macrophages from C57BL/6 mice (**L**). Cryptococcal cell dissemination, immune response and behavioral alterations (**M**). SDA: Sabouraud Dextrose Agar. MIC: Minimum inhibitory concentration. PFGE: Pulsed field gel electrophoresis. CFU: Colony forming units. BALF: bronchoalveolar lavage fluid. MPO: Myeloperoxidase activity. i.t.: intratracheal infection. PS: polysaccharide.

The synopsis of the methodology is presented in Figure 1. After determining the MIC of fluconazole on SDA, an average of five colonies from the highest MIC assay was selected for culture on SDA plates supplemented with fluconazole in a stepwise manner. The strain able to grow at the highest fluconazole concentration was chosen for the remaining tests. L27/01 strain of *C. gattii* was cultivated in high fluconazole concentrations and developed decreased drug susceptibility. After induction of reduced azole susceptibility, this phenotype was named L27/01_F_. To verify the maintenance of reduced fluconazole susceptibility, the L27/01_F_ phenotype was cultured in SDA without drug 170 times, and the MIC was determined [11] every 5 subcultures (Figure 1A–1H).

### RAPD analysis, ITS Sequencing, Pulsed field gel electrophoresis (PFGE) and ergosterol quantification

The genetic similarity between L27/01 and L27/01_F_ strains was evaluated by randomly amplified polymorphic DNA (RAPD)-PCR and pulsed field gel electrophoresis (PFGE). RAPD-PCR was performed [14] with two decamer oligonucleotides (OPA10: 5′ GTGATCGCAG 3′ and OPX17: 5′ GACACGGACC 3′). Pulsed field gel electrophoresis (PFGE) was performed according to Saracli et al. [15] with modifications. Briefly, 10^8^ cryptococcal cells and 10 mg/mL (lysing enzyme from *Trichoderma harzianum*, Sigma, St Louis, MO, USA) were used for preparation of spheroplast yeast cells. The running conditions were 100–200 s at 3.5 V for 16 h followed by 200–300 s at 4.0 V for 32 h. PFGE marker, 0.225–2.2 Mb *S. cerevisiae* chromosomal DNA (Bio-Rad) was used as size standard. Internal transcribed spacer (ITS) region 1 of L27/01 and L27/01_F_ strains was amplified using primers: ITS1 (5′TCCGTAGGTGAACCTGCGG 3′) and ITS4 (5′TCCTCCGCTTATTGATATGC 3′) [16]. The PCR products were cloned into a pCR 2.1-TOPO plasmid (Invitrogen, Calsbad, CA) and purified by using a Spin Miniprep Kit protocol (Qiagen), and then was subjected to bidirectional sequencing as described elsewhere [16], [17]. Results were submitted to a GenBank Basic Local Alignment Search Tool (BLAST). Also, the lipid evaluation of L27/01 and L27/01_F_ strains was performed using n-heptane (Sigma-Aldrich) and the reading was performed in spectrophotometer at 282 nm as described previously [8], [13] to compare the ergosterol content of L27/01 and L27/01_F_ cell membranes (Figure 1I).

### Enzymes activities

For the urease activity measurement, 10^5^ yeast cells were added to Christensen's urea broth (Difco, Detroit, U.S.A.) [18]. For the laccase activity, 10^8^ yeast cells were added to L-Dopa (Sigma) solution (20 mM) [19]. After 24 h at 37°C, absorbance was measured at 550 nm and 450 nm, respectively (Figure 1I).

### Intratracheal infection

C57BL/6 male mice/group (6 to 8 weeks old) were used in all experiments. Mice (n = 8/group) were anesthetized i.p. injection of ketamine hydrochloride (50 mg mL^−1^) and xylazine (0.02 mg mL^−1^) in sterile saline, then inoculated i.t. with 30 µL of 1×10^6 ^CFU/animal of L27/01 or L27/01**_F_** strains or PBS only (control). Animals were monitored twice daily for survival. To verify *in vivo* resistance, fluconazole (10 mg/kg/day) was administered daily i.p., from 5 to 11 days post infection (d.p.i.) (Figure 1J) and lungs were removed for the determination of colony forming units (CFU) as described below.

### Capsule analysis

For capsule induction, L27/01 and L27/01**_F_** cells were grown at 30°C in minimal medium (glucose, 16 mM; MgSO_4_, 10 mM; KH_2_PO_4_, 29.4 mM; glycine, 13 mM; pH 5.5) for 5 days. The capsule sizes of 100 yeast cells of L27/01 and L27/01**_F_** (in India ink) were measured using ImageJ 1.40 g (National Institutes of Health (NHI), Bethesda, MD) with an optical microscope (AXIOPLAN, Carl Zeiss). Cells were also observed with a QUANTA scanning electron microscope (FEI, Oregon, USA) (Figure 1K).

Polysaccharides (PS) released from L27/01 or L27/01**_F_** cells were isolated from culture supernatants by filtration and extraction of capsular components with DMSO. Ultrafiltration using an Amicon (Millipore, Danvers, MA) ultrafiltration cell (cutoff = 100 kDa) was performed as described previously [20]. The final PS was quantified and surface-associated capsular PS was isolated from yeast cells [21]. Further, yeast cells (10^6^) were incubated for 1 h with mAb 18B7 (10 µg/ml). Then, cells were incubated with FITC-labeled goat anti-mouse IgG (Fc specific) antibody (Sigma) for 30 min at 37°C [22].

The effective diameter and polydispersity of PS preparations were measured by QELS in a 90Plus/BI-MAS multi-angle particle sizing analyzer (Brookhaven Instruments Corp., Holtsville, NY) as described by Frases et al. [23]. Zeta potential (), particle mobility and shift frequency of polysaccharide samples were calculated in a Zeta potential analyzer (ZetaPlus, Brookhaven Instruments Corp., Holtsville, NY) [24].

### RNA extraction, reverse transcription and real-time PCR

Total RNA from L27/01 and L27/01**_F_** strains was extracted with RNeasy kit (Qiagen, Germany), and RT-PCR was performed using the enzyme MMLV (Promega, Madison, WI, USA). cDNA was used to determine *CAP59, CAP64, MDR1, ARF-1, ERG11, UXS-1* levels by real-time PCR using 200 nM of specific primers and SYBR Green Master Mix (Applied Biosystem, USA, 10 µL) (Figure 1I). The primers used (available in Table S1) were designed according to the DNA sequences available from the *C. gattii* database from the Broad Institute (http://www.broadinstitute.org/annotation/genome/cryptococcus_neoformans_b/MultiHome.html). Analyses were performed using the delta-delta Ct method and normalized using the *C. gattii* actin gene.

### Phagocytosis assay, Intracellular Proliferation Rate, measurement of ROS production by macrophages and enzymatic activity of the antioxidant system

The phagocytosis assay was performed as described previously [1] to assess the influence of PS capsules from L27/01 and L27/01_F_ strains on phagocytosis, on the intracellular proliferation rate (IPR) in murine peritoneal macrophages from C57BL/6 mice, and on the ability to neutralize ROS during the phagocytosis assay. Briefly, murine macrophages (2×10^5^ cells/mL) were obtained 3 days post thioglycollate (3%) injection and maintained in RPMI 1640 supplemented with 10% fetal bovine serum. Macrophages were immediately infected with L27/01 or L27/01_F_ yeast cells (6×10^4^ cells/mL) opsonized with 10% murine serum, with or without PS (10 mg/L), and incubated for 3 or 27 h at 37°C under 5% CO_2_ for phagocytic index, intracellular proliferation rate (IPR) determination, ROS quantification, superoxide dismutase (SOD) and peroxidase (PER) measurement. The phagocytic index was calculated as the percentage of cells with internalized *C. gattii* after 27 h, while the IPR was calculated as the quotient of the intracellular yeast cell numbers at 27 h (time point which was the maximum intracellular yeast number) and 3 h [1] (Figure 1L).

2′,7′-dichlorofluorescein diacetate (DCFH-DA; Invitrogen, Life Technologies, Carlsbad, CA, USA) was used for ROS quantification and the fluorescence was measured with a fluorometer (Synergy 2 SL Luminescence Microplate Reader; Biotek) [13]. A cell-free extract from *C. gattii*-infected macrophages or fungus or PS capsule alone (control groups) was obtained for SOD and PER activities [13].

### Colony Forming Units (CFU), myeloperoxidase activity, cytokines and chemokines levels and histopathology

Groups of C57BL/6 male mice (n = 8/group) were anesthetized i.p., then inoculated i.t. with 1×10^6 ^CFU/animal of L27/01 or L27/01_F_ strains or PBS only (control) to obtain bronchoalveolar lavage fluid (BALF), lungs and brain at 0.5, 1, 7, 15, or 30 days post inoculation. Organ homogenates were plated onto SDA to determine the CFU. The BALF was obtained for neutrophils and mononuclear leukocytes counts [25] (Figure 1M). Lung fragments were removed for the myeloperoxidase activity [26] assay and quantification of cytokines and chemokines (TNF-α, IFN-γ, CXCL1/KC and IL-10). Also, lungs and brain were stained with hematoxylin-eosin (HE) and Grocott’s (Easy Path) (Figure 1M).

### Behavioral analysis

The SHIRPA protocol for behavioral and functional assessment of neurological diseases was used (Figure 1M). The tasks are grouped into five functional categories: neuropsychiatric state, motor behavior, autonomic function, muscle tone and strength, and reflex and sensory function (available in Table S2) [27], [28]. The mice were examined daily and the score for each functional categories was calculated as the total of the evaluated parameters according to Lackner et al. [27] and Pedroso et al. [28] using the EpiData 3.1 software.

### Statistical analysis

Results are shown as means ± SEM. Statistical analysis of all data were performed using GraphPad Prism version 5.0 with p<0.05 considered significant. Survival curve was plotted by Kaplan-Meier analysis and results were analyzed using the log rank test. Weight variation was analyzed with Wilcoxon Rank-Sum Test. The results of *MDR1, CAP59* and *CAP64* genes expression, capsule size, ergosterol, enzymes and CFU/g organ or BALF were analyzed by analysis of variance (ANOVA) and the nonparametric Friedman test, Student’s t test. Also, the results of phagocytosis assay, intracellular proliferation rate, measurement of ROS production by macrophages and enzymatic activity of the antioxidant system, myeloperoxidase activity and quantification of cytokines and chemokines were analyzed by analysis of variance (ANOVA) and the nonparametric Friedman test, Student’s t test. Statistical analyses were carried out using 90Plus/BI-MAS software for effective diameter and polydispersity (Brookhaven Instruments Corp., Holtsville, NY). Zeta Plus software was used for Zeta potential, (Brookhaven Instruments Corp., Holtsville, NY). SHIRPA data were analyzed with Wilcoxon Rank-Sum Test (28).

## Results

### L27/01 and L27/01_F_ show differences in susceptibility to fluconazole

**Figure 2:**
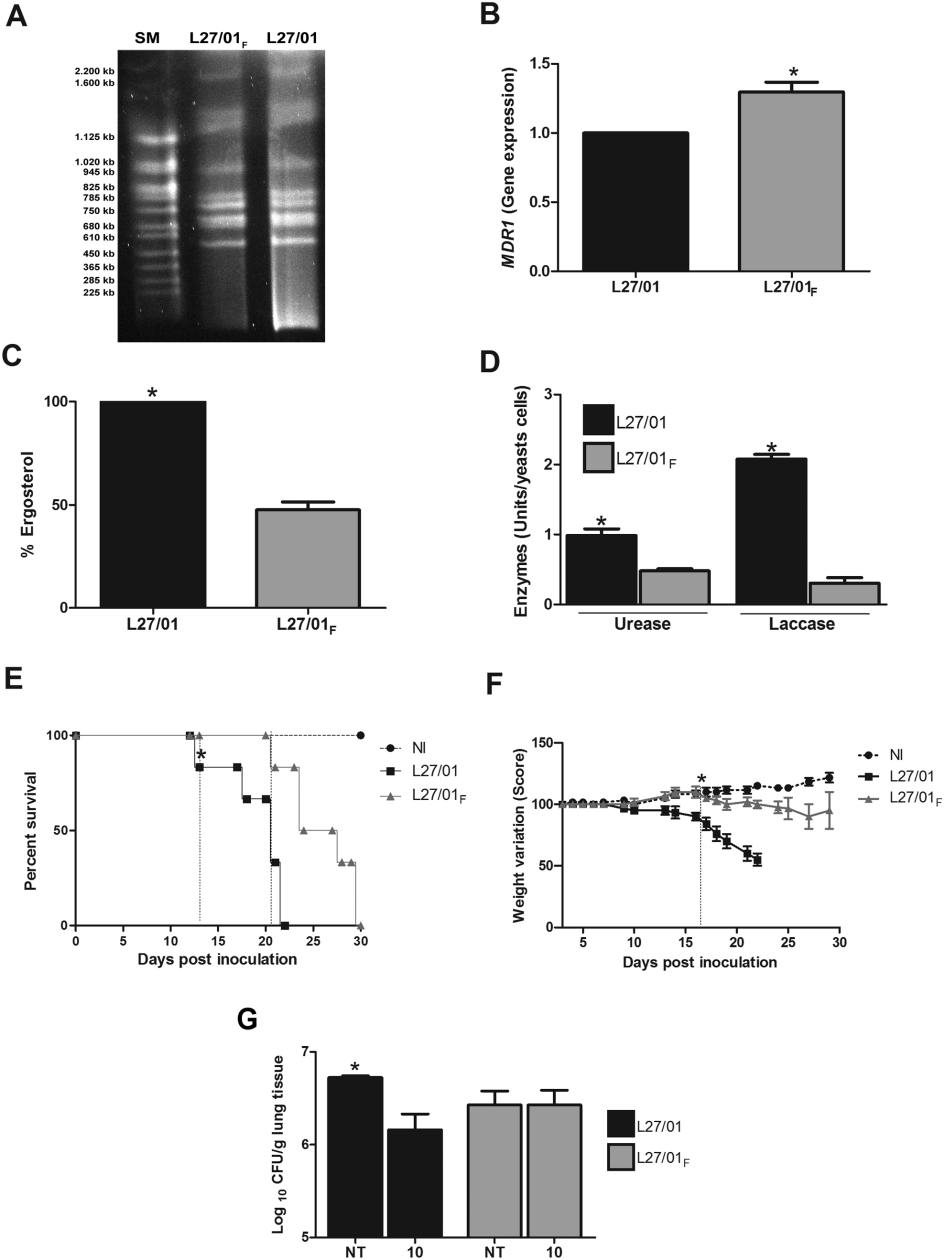
L27/01 developed reduced susceptibility to fluconazole with overexpression of the *MDR1* gene, but the parental strain is more virulent than L27/01_F_ in mice. Pulsed field gel electrophoresis (PFGE) patterns of L27/01_F_ and L27/01 and strains on 1% PFGE certified agarose (lanes 2 and 3, respectively). Lane 1 show PFGE Size Marker (SM), 0.225–2.2 Mb *S. cerevisiae* chromosomal DNA *Saccharomyces cerevisiae* used as size standard (**A**). *MDR1* gene expression in L27/01 and L27/01_F_ strains (**B**). Ergosterol levels of L27/01 and L27/01**_F_** strains (**C**). Urease and Laccase activities of L27/01 and L27/01**_F_** strains: one enzymatic unit was calculated per 10^5^ and 10^8^ cells of *C. gattii* for urease and laccase activity, respectively, after 24 hours (**D**). Eight mice per group were inoculated by the intratracheal route with 10^6^ cells of L27/01 or L27/01**_F_**. Animals administered with PBS represent the non-infected (control) group. Animals were monitored daily. Survival curve (**E**). Weight variation (Score) (**F**). Each 1 (one) gram received or lost corresponds to 10 points added or subtracted, respectively. *The vertical dotted line indicates a significant difference from the appointed day. CFU/g of lungs (**G**) from animals infected with 1×10^6^ cells of L27/01 or L27/01**_F_** strains treated i.p. with fluconazole at 10 mg/kg/day (n = 6). NI: not infected group. NT: non treated group. Black bars refer to L27/01 (▪) and grey bars refer to L27/01**_F_** (▴). Data represent the mean of three independent experiments in triplicate. *P<0.05 was considered to be significant.

Initially, 12 strains of *C. gattii* were used in susceptibility tests with fluconazole (by microdilution and supplemented solid medium) and with amphotericin B (by the microdilution method) (data not shown). During the tests, we chose an average of five colonies of the strain that was able to grow at the highest concentration of fluconazole for successive subcultures every 48 hours with increasing concentrations of fluconazole: 32; 64; 128 and 256 µg/mL. As the growth was interrupted at 128 µg/mL, we reduced the drug range concentration in solid medium to: 64; 70; 75; 80; 85; 90; 95; 100; 105 and 110 µg/mL. Thus, growth was observed up to a concentration of 95 µg/mL since the growth was interrupted at 100 µg/mL. L27/01 strain was chosen due to growth at the highest fluconazole concentration. The minimum inhibitory concentrations (MICs) for fluconazole against the L27/01 strain varied from 8.0 to 16.0 µg/mL and for amphotericin B it was 0.06 µg/mL. This strain was chosen to be tested in the other experiments and was cultured in solid medium with increasing concentrations of fluconazole until growth ceased at 100 µg/mL. Colonies grown at 95 µg/mL were maintained in this concentration (Figure 1C–G), and the phenotype was named L27/01_F_ (Figure 1G). This procedure showed significantly reduced susceptibility to fluconazole which persisted until the 170^th^ subculture in drug-free medium as confirmed by the microdilution (MIC = 128 µg/mL). For amphotericin B, the MIC increased from 0.06 to 1 µg/mL, demonstrating cross-resistance with fluconazole. Phenotypic examination revealed that L27/01 strain produced mucous colonies, while the stress caused by fluconazole (L27/01_F_ strain) led to dry colonies (data not shown). RAPD (data not shown) and PFGE (Figure 2A) revealed identical banding patterns between L27/01 and L27/01_F_, which were clustered on the same clade with 100% similarity. We performed the sensibility test of the two strains to different temperatures and we verified no diferences between them to high temperatures (data not shown), corroborating the results of PFGE. The DNA sequence of the ITS1 region PCR products (555 bp) of L27/01 and L27/01_F_ strains were analyzed from the BLAST search and corresponded to *C. gattii* isolate deposited in GenBank (accession n°. JN939462.1) with 100% identity and 100% query coverage. Also, the alignment of these sequences displayed 100% similarity between L27/01 and F strain (data not shown). Furthermore, L27/01 and L27/01_F_ strains were subjected to RT-PCR to test for expression of genes related to efflux pumps (*AFR1* and *MDR1*) or ergosterol synthesis (*ERG11*). The L27/01_F_ strain overexpressed the *MDR1* gene (Figure 2B) (p<0.05), but expression of *ARF1* and *ERG11* genes was similar in L27/01 and L27/01_F_ strains (data not shown). Ergosterol content was 50% lower (p<0.05) in the L27/01_F_ strain compared to L27/01 (Figure 2C).

### L27/01 *C. gattii* is more virulent than L27/01_F_

Urease and laccase (Figure 2D) activities were significantly higher for the L27/01 strain (p<0.05). Additionally, mice infected with the L27/01 strain survived for 22 days post-inoculation and mice inoculated with or L27/01**_F_** succumbed after 30 days (p<0.05) (Figure 2E). Weight loss was significantly higher after infection with L27/01 (p<0.05) (Figure 2F).

### Reduced susceptibility to fluconazole in strain L27/01_F_ *in vitro* was associated with *in vivo* antifungal resistance

Interestingly, animals infected with L27/01 and treated with 10 mg/kg/day of fluconazole showed a significant (p<0.05) reduction of fungal burden in the lungs, while animals infected with L27/01**_F_** presented no reduction in CFU compared with untreated infected mice (Figure 2G).

Reduced virulence of *C. gattii* was associated with alterations in the polysaccharide capsule: reduced susceptibility to fluconazole impairs GXM synthesis and secretion, but does not block polysaccharide assembly.

**Figure 3:**
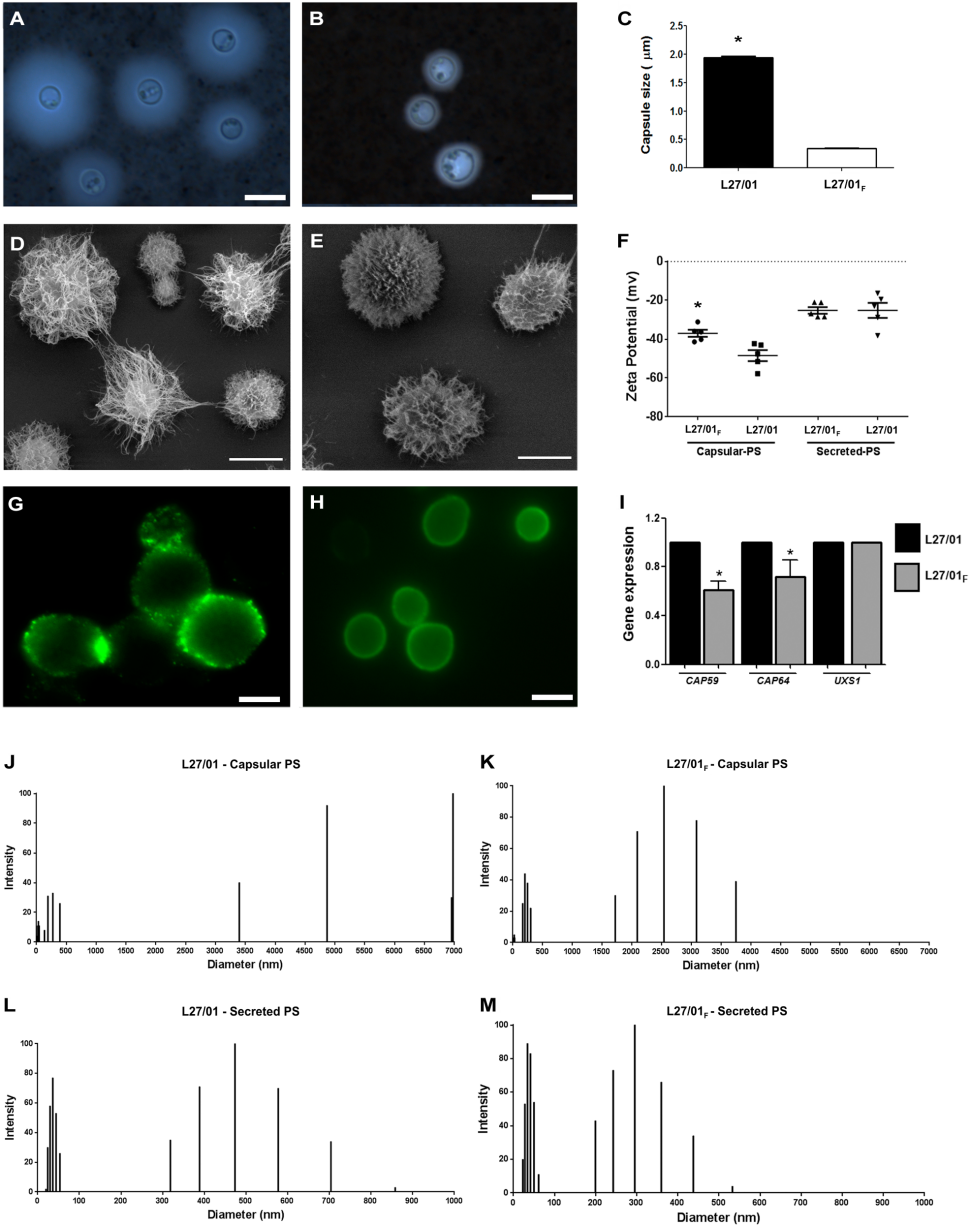
Reduced susceptibility to fluconazole leads to very low *CAP59* and *CAP64* gene expression and affects physical properties of the GXM capsule. Microscopic visualization with India ink stain of L27/01 (**A**) and L27/01**_F_** strains (**B**). Capsule size of L27/01 and L27/01**_F_** strains (**C**). SEM analysis of L27/01 (**D**) and L27/01**_F_** (**E**) strains. Zeta potential of capsular and secreted PS of cells (**F**). Cell suspensions were analyzed with an AXIOPLAN (Carl Zeiss) fluorescence microscope. Images were processed using ImageJ. Staining of L27/01 (**G**) and L27/01**_F_** (**H**) strains with mAb 18B7 showing differences in epitope presentation in the PS capsule between strains. Ratio of gene expression of *CAP59*, *CAP64* and *UXS1* to actin (**I**). Size distribution of PS fibers from capsular (**J** and **K**) and exo-PS samples (**L** and **M**) of L27/01 and L27/01**_F_** strains, respectively. Bar = 10 µm (A) and (D). Bar = 5 µm (B), (E), (G) and (H). *P<0.05 was considered to be significant.

Since we found distinct profiles of disease caused by the L27/01 and L27/01**_F_** strains, we further investigated whether the reduction of susceptibility to fluconazole affects the main virulence factor of the genus *Cryptococcus*: the polysaccharide capsule. L27/01**_F_** strain showed reduced ability to form a polysaccharide capsule compared to the L27/01 strain (Figure 3A–C). Genes related to capsule (*CAP59* and *CAP64*) were quantified by RT-PCR; *CAP59* and *CAP64* expression were significantly (p<0.05) higher in the L27/01 strain (40% and 30%, respectively) (Figure 3I). However, the expression of a gene related to polysaccharide assembly (*UXS1*) was similar between strains (Figure 3I). No significant differences in extracellular polysaccharide levels were apparent (2.3 mg/mL for the L27/01**_F_** strain and 2.5 mg/mL for L27/01). DMSO extracts of whole cells revealed differences between fractions (5.3 mg/ml for L27/01 and 2.5 mg/ml for L27/01**_F_** strain), which is consistent with results from India ink (Figure 3A–B) and scanning electron microscopy (Figure 3D–E).

### Fluconazole affects physical and structural properties of the L27/01_F_ strain capsule

Considering that the physical properties of the cryptococcal capsule are related to binding by monoclonal antibodies, we examined the fluorescence pattern of the 18B7 monoclonal antibody (IgG1) bound to the L27/01**_F_** and L27/01 strains (Figure 3G–H). The results showed differences in epitope presentation in capsular-PS for both strains with lower fluorescence intensity for L27/01**_F_** strain, revealing differences in opsonine patterns that may influence phagocytosis.

Hydrodynamic properties of L27/01**_F_** and L27/01 PS were calculated from dynamic light scattering measurements. The L27/01 strain showed a longer PS capsule size distribution with a maximum size of 7000 nm; L27/01**_F_** cells presented a polydispersed size distribution shorter than L27/01 (Figure 3J–K). For secreted PS, L27/01**_F_** and L27/01 cells showed no significant differences in effective diameter (Figure 3L–M). Conversely, an important finding is that capsular PS differed in Zeta potential between the parental and L27/01**_F_** strains (Figure 3F). However, secreted PS did not show mobility differences between the two strains (Figure 3F).

### L27/01_F_ strain is more internalized by macrophages

**Figure 4:**
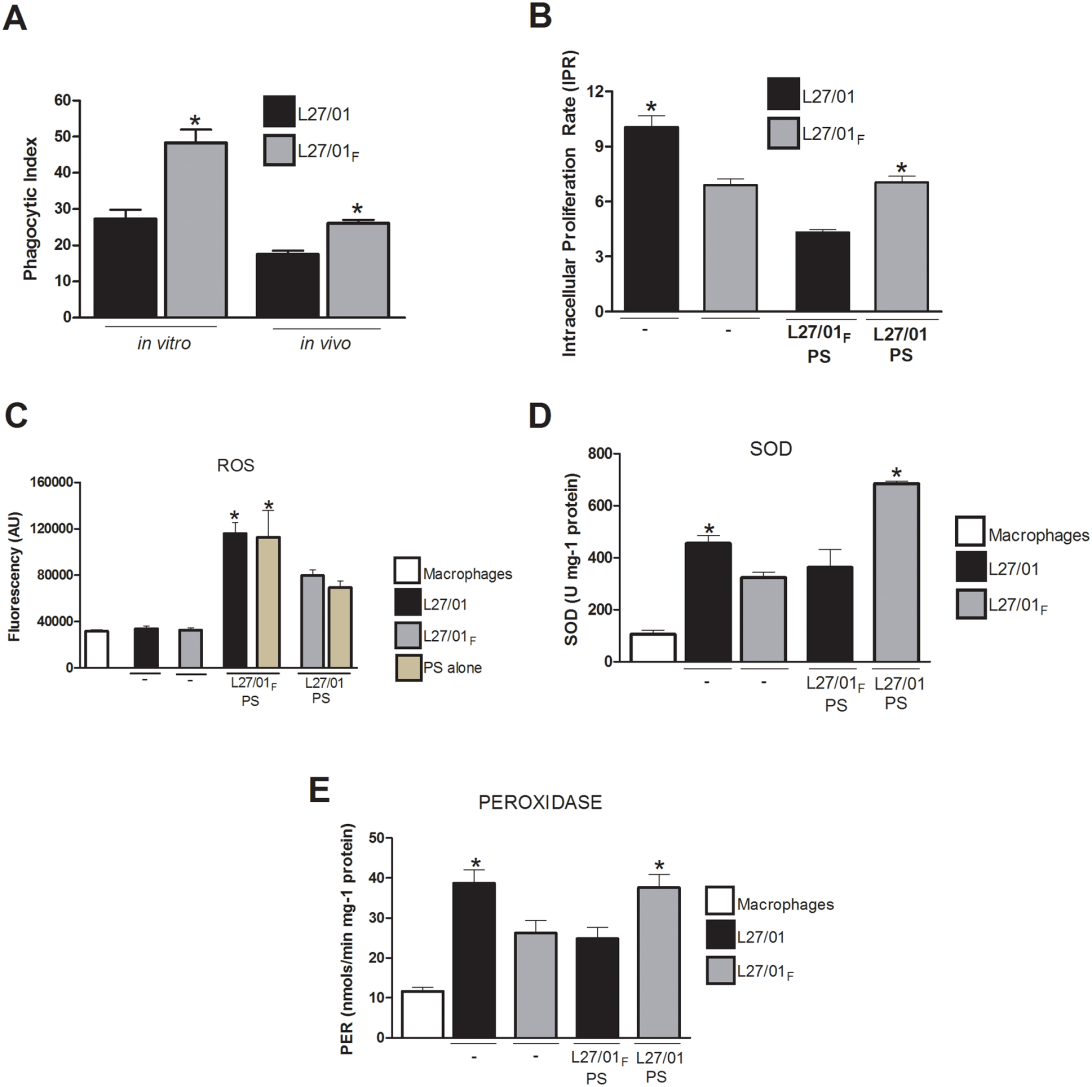
The L27/01_F_ strain is more readily internalized by macrophages, but is not able to proliferate as well as the L27/01 strain. The phagocytosis assay was performed *in vivo* by counting internalized yeast cells in BALF cells, and *in vitro* with murine peritoneal macrophages infected with *C. gattii*. The influence of L27/01 and L27/01**_F_** polysaccharide capsules (L27/01 PS and L27/01**_F_** PS) on intracellular proliferation rate (IPR), reactive oxygen species, superoxide-dismutase (SOD) and peroxidase (PER) activities was also evaluated. White bars refer to macrophages (control), black bars to L27/01, grey bars to L27/01**_F_** and beige bars to PS alone without yeasts. Phagocytic index (**A**). IPR (**B**). Reactive oxygen species expressed in arbitrary units of fluorescence (AU) (**C**). SOD (U mg^−1^ protein) (**D**) and PER (nmol/min mg^−1^ protein) (**E**) activities after phagocytosis. The data are presented as the mean ± S.E.M. of two independent experiments in triplicate. *P<0.05. Symbols indicate absence (–) of PS in the phagocytosis assay.

Considering the differences in PS capsule between the two strains and the well characterized anti-phagocytic properties of GXM in previous studies, the phagocytosis assay was performed *in vivo* by counting internalized yeasts in BALF cells and *in vitro* with murine peritoneal macrophages infected with *C. gattii*. The influence of the polysaccharide of L27/01 and L27/01**_F_** strains on the phagocytic index (Figure 4A) and intracellular proliferation rate (IPR) (Figure 4B) were determined. The results demonstrated similar profiles *in vitro* and *in vivo*; the L27/01**_F_** strain was more susceptible to internalization than the L27/01 strain (p<0.05). The L27/01 strain demonstrated a greater ability to proliferate inside macrophages and the proliferation was reduced when the L27/01**_F_** polysaccharide capsule was added to the assay (p<0.05).

In addition, we evaluated whether fluconazole altered the antioxidant properties of the polysaccharide capsule. Reactive oxygen species (ROS) were determined in the supernatant from the phagocytosis assay with the L27/01 or L27/01**_F_** polysaccharide (PS) capsule (Figure 4C). The results demonstrated that ROS (Figure 4C) production was significantly higher (p<0.05) when the macrophages were infected with the L27/01**_F_** PS as well as when it was added to the culture of the L27/01 strain. Alternately, the L27/01 PS capsule added to the L27/01**_F_** strain improved neutralization of ROS. These results show that stress to fluconazole compromises resistance to ROS.

For further investigation of how PS may neutralize ROS inside macrophages, we determined the activities of SOD and PER in yeast cells after 3 h of the phagocytosis assay. The results show that SOD activity was significantly higher for the L27/01 strain (p<0.05) and for the L27/01**_F_** strain plus L27/01 PS (p<0.05) (Figure 4D). When the total PER activity (Figure 4E) was measured, the L27/01 strain and the L27/01 PS capsule showed higher (p<0.05) PER activity than the L27/01**_F_** strain and its polysaccharide capsule.

### Reduced susceptibility to fluconazole impairs translocation to the CNS

**Figure 5:**
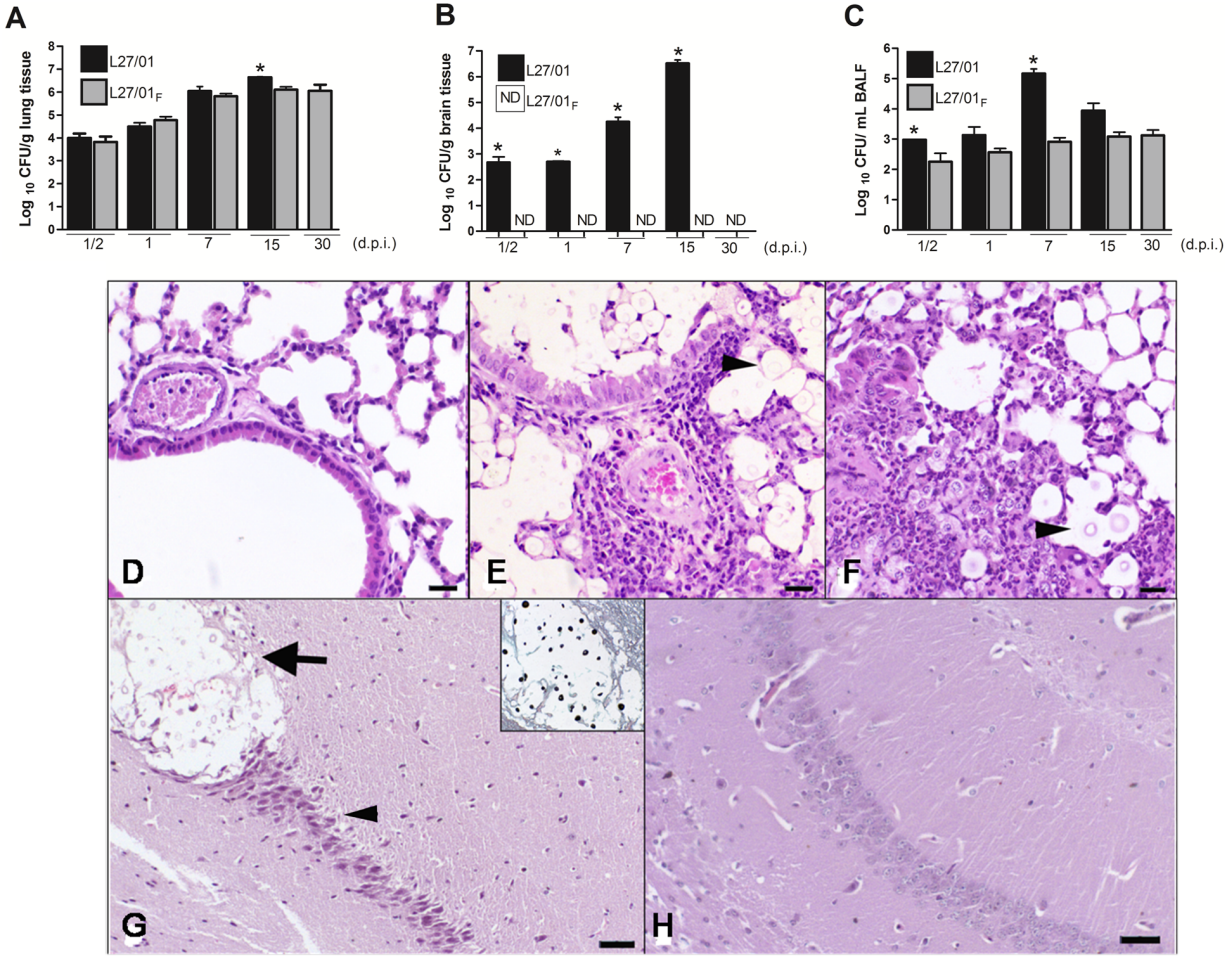
Reduced susceptibility to fluconazole impairs *C. gattii* migration to the CNS. Lungs (**A**), brain (**B**), and Bronchoalveolar lavage fluid (BALF) (**C**) were removed (n = 8), homogenized, diluted and plated onto Sabouraud dextrose agar for measurement of fungal burden 0.5, 1, 7, 15, and 30 days post-infection with 10^6^ cells of L27/01 or L27/01**_F_** strains. ND: non-detected. Black bars refer to L27/01 and grey bars refer to L27/01**_F_**. *Data represent the mean of two independent experiments in triplicate. P<0.05 was considered to be significant. (**D–H**) Histopathological sections stained with H&E of lung and brain 15 days post-inoculation. Lungs of control mice (**D**). Lungs of mice inoculated with L27/01 strain showing mild peribronchiolar and moderate perivascular neutrophilic and histiocytic inflammatory infiltrate associated with numerous yeast cells in the peribronchiolar and alveolar lumen (**E**). Lungs of mice inoculated with L27/01**_F_** strain, with intense diffuse inflammatory infiltrate, neutrophils and macrophages around few yeast cells (head arrow) (**F**). Bar = 40 µm. Hippocampi of mice inoculated with L27/01 strain showed cryptococcoma (arrow), neuronal degeneration and death (head arrow). *Cryptococcus gattii* yeasts in cryptococcoma were stained with Groccot’s stain (in detail) (**G**). Hippocampi of mice inoculated with L27/01**_F_** strain showed unaltered structure (**H**). Bar = 20 µm.

Since different profiles were found in the phagocytosis assay and since the fungal translocation to the CNS was previously associated with the ability to survive in the macrophage intracellular environment, we verified the kinetics of the infection caused by the two strains. After, 0.5, 1, 7, or 15 days post-inoculation with yeast cells, the fungal burden in lungs and brain were significantly higher in mice infected with the L27/01 strain (Figure 5A and B). The ability of the L27/01**_F_** strain recovered from the organs to grow at higher concentrations of fluconazole was maintained, as verified in medium supplemented with this drug (data not shown). The L27/01**_F_** strain did not disseminate to the brain until 30 days post-inoculation, while the L27/01 strain colonized the CNS within 12 hours (Figure 5B). Analysis of bronchoalveolar lavage fluid (BALF) (Figure 5C) revealed that the fungal burden of L27/01 strain increased within 7 days post-inoculation (p<0.05), while in the L27/01**_F_** strain the burden remained lower in the extracellular space. These results were confirmed in three independent experiments in triplicate.

In the histopathological analysis, yeasts varying from 5 to 30 µm in size were observed free within the cytoplasm in the bronchial, bronchiolar and alveolar lumen. Infiltrate resulting from mild to intense inflammation was composed of neutrophils and macrophages, sometimes forming granulomas (Figure 5E and F). Thickening of the alveolar walls was also observed. The intensity of lesions in the lung differed between strains 15 days post-infection, being mild in mice inoculated with the L27/01 strain and moderate to intense in mice inoculated with the L27/01**_F_** strain. In brain tissue, cryptococcomas, characterized by a focal mass containing multiple grouped yeast cells associated with the destruction of the neuropil, were only observed in the hippocampus of mice infected with the L27/01 strain; in other areas, no alteration was detected (Figure 5G). Although cryptococcomas were not associated with inflammatory infiltration, degeneration and neuronal death were observed in the hippocampus (Figure 5G).

### L27/01_F_ strain increases the recruitment of host cells to the lungs, but decreases production of IFN-γ, TNF-α, IL-10 and CXCL1/KC

**Figure 6:**
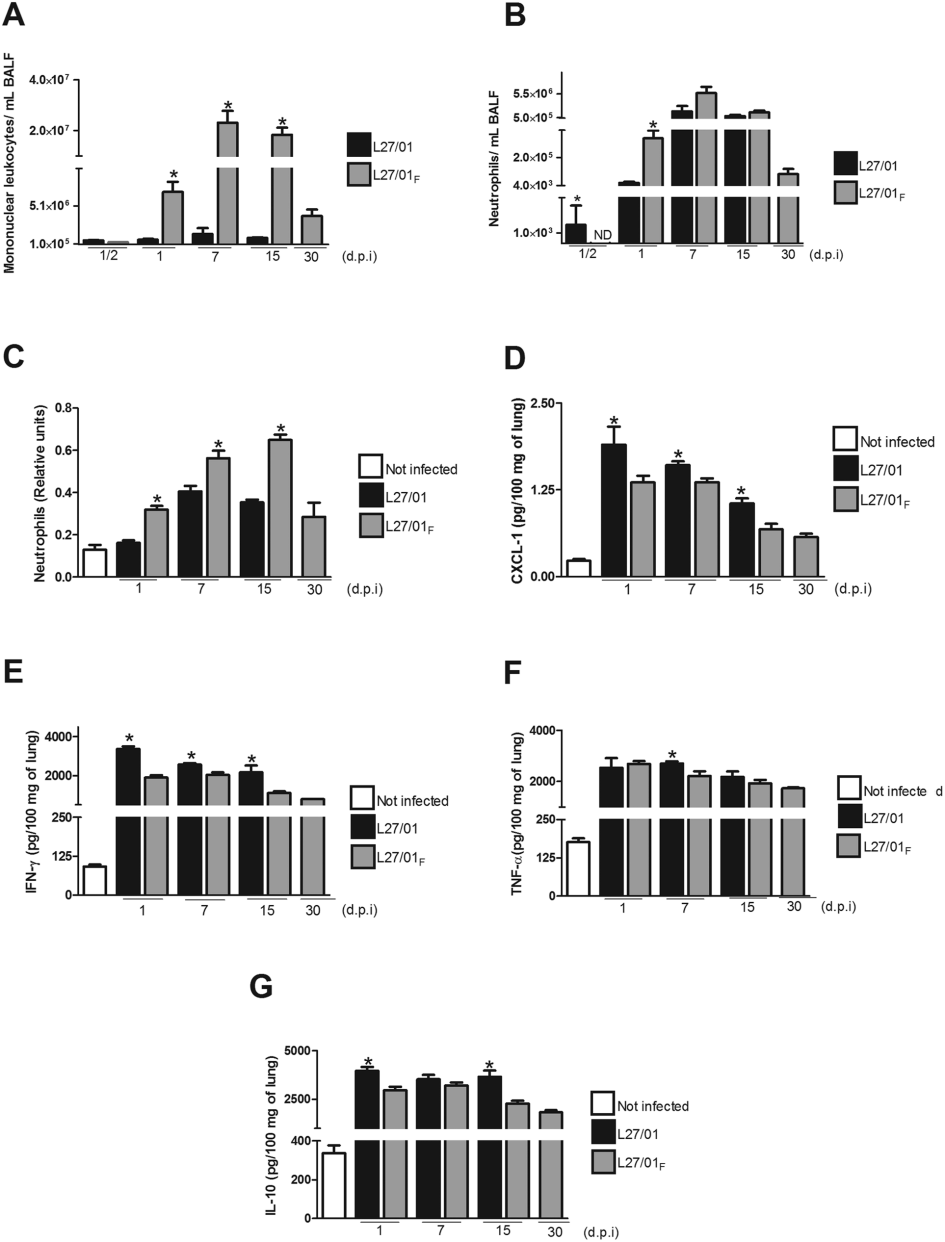
L27/01_F_ increases recruitment of host cells to the lungs, but decreases cytokine production. L27/01 and L27/01**_F_** strains elicit different inflammatory response in C57/BL6 mice. Mice (n = 8) were inoculated i.t. with 10^6^ yeast cells of L27/01 or L27/01**_F_** strains. After 1, 7, 15 or 30 days post infection, the mice were euthanized and mononuclear leukocytes (**A**) and neutrophils (**B**) in bronchoalveolar lavage fluid (BALF) were quantified. Neutrophilic infiltrate in the lungs was also determined as MPO activity and expressed as relative number of neutrophils (**C**). Levels of CXCL1/KC (**D**), IFN-γ (**E**), TNF-α (**F**) and IL-10 (**G**) are expressed in pg per 100 mg of lung. White bars refer to not infected (NI) animals, black bars to animals infected with the L27/01 strain, and grey bars to animals infected with the L27/01**_F_** strain. The results are presented as the mean ± SEM. Each experiment was performed in triplicate. *P<0.05 was considered to be significant.

Mononuclear leukocytes and neutrophils were more recruited to the lungs by the L27/01**_F_** strain than the L27/01 strain (p<0.05) (Figure 6A–B). The MPO activity in the lungs (Figure 6C) confirmed higher neutrophil influx with the F strain (p<0.05).

The L27/01 and L27/01**_F_** strains also triggered different levels of cytokine production. Levels of CXCL1/KC (Figure 6D), IFN-γ (Figure 6E), TNF-α (Figure 6F), and IL-10 (Figure 6G) in the lung 1, 7, 15 and 30 days post-infection were significantly higher (approximately 30%, 40%, 19% and 36%, respectively) (p<0.05) in mice infected with the L27/01 strain, particularly 1 to 7 days post-infection.

### L27/01 leads to significant behavioral alterations associated with neurocryptococcosis

**Figure 7:**
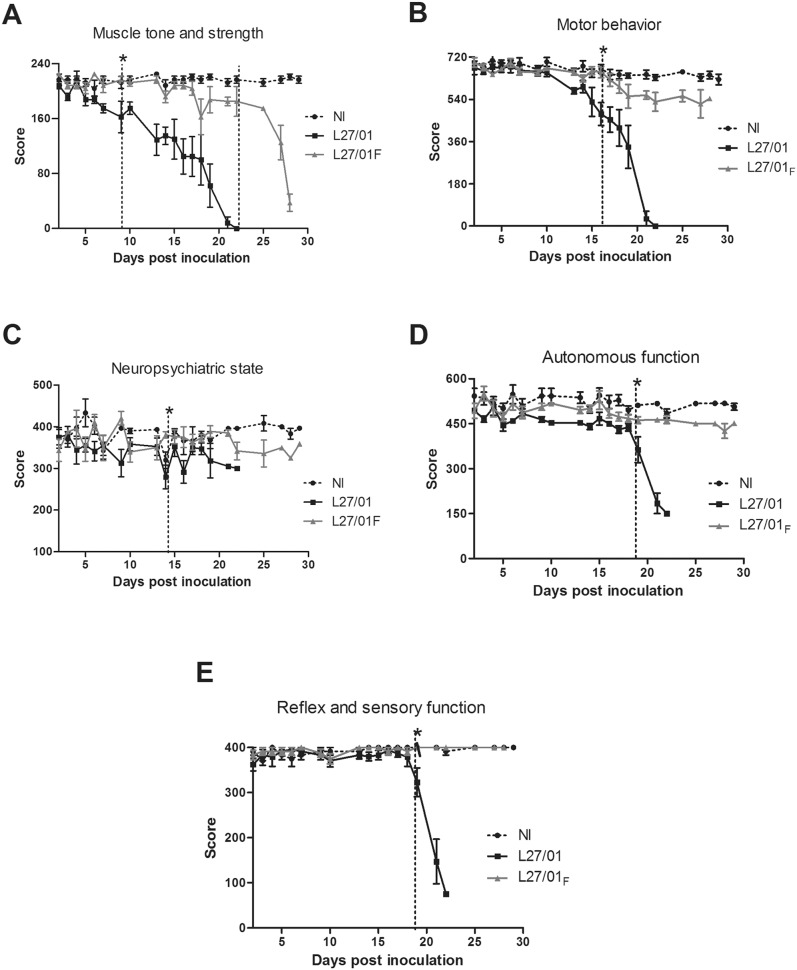
L27/01 alters the clinical manifestations of cryptococcosis. Assessment of behavioral performance of not infected (NI) mice and those infected with L27/01 (▪) or L27/01**_F_** (▴) strains in five distinct functional categories of the SHIRPA battery (n = 6) (**A**–**E**). Mice were monitored daily for: muscle tone and strength (**A**); motor behavior (**B**); neuropsychiatric state (**C**) autonomous function (**D**); reflex and sensory function (**E**) (n = 6). The results are presented mean ± SEM of eight animals in each group. Scores of the infected groups were compared with the uninfected group by the Wilcoxon matched test. *The vertical dotted line indicates a significant difference from the appointed day. P<0.05 is considered to be significant.

Mice infected with L27/01 or L27/01**_F_** strains were assessed using the SHIRPA protocol. The results demonstrated significant changes (p<0.05) in the behaviors of the two infected groups. In the L27/01 group, muscle tone and strength (Figure 7A) were altered 9 day post-inoculation (p<0.05), while this clinical function was altered in the L27/01**_F_** group only 22 days post-inoculation. Motor behavior (Figure 7B) was significantly altered after 16 days for the L27/01 strain (p<0.05). Neuropsychiatric state (Figure 7C) was significantly altered after 14 days for the L27/01 strain (p<0.05). Autonomous (Figure 7D), reflex, and sensory function (Figure 7E) were altered 19 days post-infection with L27/01 cells. Motor behavior, neuropsychiatric state, autonomous function, reflex and sensory functions were not altered in mice infected with the L27/01**_F_** strain.

## Discussion

*Cryptococcus gattii* isolates are frequently reported as less susceptible to azoles than other *Cryptococcus* species [29]–[31]. Based on the Epidemiological Cutoff Values (ECVs) defined by Espinel-Ingroff et al. [30], L27/01_F_ strain showed the ability to develop secondary resistance to fluconazole because the higher MICs (increased from 8–16 µg/mL to 128 µg/mL) persisted until the 170^th^ repeated culture in drug free medium. RAPD and PFGE analysis revealed identical banding patterns between L27/01 and L27/01_F_. According to Torres et al. [32], aneuploid strains can show increased sensitivity to high temperatures, but we verified no diferences between them to high temperatures (data not shown) in sensibility test to temperature. Although cultivating *Cryptococcus* in fluconazole supplemented media may also generate fluconazole-heteroresistant clones [33]–[35], L27/01_F_ may not be a heteroresistance phenotype, since the higher MIC values persisted after 170 subcultures in drug free medium. Heteroresistance is also a phenomenon present in *Candida albicans* [36], [37].

Interestingly, the reduced susceptibility to fluconazole was confirmed in the *in vivo* tests, as this drug was unable to reduce the CFU in the lungs of animals infected with L27/01_F_. Additionally, environmental isolates of fluconazole-resistant *C. gattii* have also been reported, demonstrating the possibility of primary resistance to fluconazole [38]. The data regarding the influence of fluconazole in *C. gattii* virulence are still scarce, but some evidence has been reported for *C. neoformans* [33], [39]. Special attention should be directed to prophylaxis with fluconazole, which increases the possibility of cross-resistance to other drugs [40], as we demonstrated for amphotericin B in the L27/01_F_ strain. The reduced ergosterol levels in the L27/01_F_ strain probably caused the cross-resistance to amphotericin B, confirming previous data [8]. On the other hand, overexpression of the *MDR1* gene was the probable mechanism of significant decreased susceptibility to fluconazole in the L27/01_F_ strain, increasing efflux to the extracellular environment. Different from our data, Gast et al. [41] found overexpression of *ERG11* gene as responsible for reduced susceptibility to fluconazole in *C. gattii* strains. Overall, we think that the mechanisms of acquired resistance may be strain-dependent and *MDR1* was prominent in our study, with no role developed by *ERG11*, since the ergosterol levels were significantly reduced in L27/01_F_.

The less virulent phenotype of L27/01_F_ was associated with lower activities of urease and laccase [18], [19], probably compromising its inability to disseminate to the CNS together with poor capsule formation. The lower expression of *CAP59* and *CAP64* genes confirmed these results. Although the exact role of the encoded Cap proteins remains unknown, it has been suggested that *CAP59* plays a role in the trafficking of GXM PS molecules [42], whereas proteins encoded by *CAP64* are involved in the acetylation of GXM [43]. The *CAP64* gene is reportedly essential for virulence [44], [45]. The data allowing for the description and characterization of less susceptible *C. gattii* are still scarce, but some data on *C. neoformans* suggests that fluconazole and other drugs may promote capsule shedding, leading to poorly encapsulated or acapsular cells [46], but without a correspondence in murine model since most of these studies focus in vitro analyses. Our findings may have clinical implications because a large capsule may facilitate fluconazole penetration into the yeasts due to its more hydrophilic profile [47]. Altogether, a relationship between the ergosterol biosynthetic pathway and the virulence of cryptococcal cells is plausible [48].

L27/01 cells were able to modulate the recruitment of host cells to the lung. The virulence of the L27/01 strain was higher in this murine model owing to intracellular replication and lack of susceptibility to macrophages, most likely due to the properties of its PS capsule [47], [49]. According to Johnston and May [50], one speculative possibility is that phagocytosis of *Cryptococcus* by macrophages can fail due to capsular PS shedding from the fungal cell, causing the internalization of only PS fractions and releasing of cryptococcal cell. The similarity between strains in the expression of the *UXS1* gene, which is related with the incorporation of PS fibers to the capsule [51] showed that L27/01_F_ is able to increase the PS content of the cell, corroborating the results found for intracellular proliferation, ROS and antioxidant enzymes: in these assays, addition of L27/01 PS to the L27/01_F_ strain provided protection. Fluconazole also reduced the negative charge and most likely the electrostatic repulsion between the L27/01_F_ strain and macrophages, probably increasing phagocytosis [46].

It is still unclear how the PS capsule neutralizes oxidative bursts and promotes fungal survival inside macrophages, although these properties have been mentioned previously [47]. In our study, the higher susceptibility of the L27/01**_F_** strain to ROS was associated with impairment of the antioxidant properties of the PS, which showed lower SOD and PER activities during phagocytosis. These data provide new insights into the influence of antifungal drugs on the virulence factors of *C. gattii*, demonstrating a connection between fluconazole resistance and the antioxidant system.

The murine model developed in this study clearly mimics the natural route of infection and revealed the ability of the L27/01 strain to disseminate to the CNS. This was confirmed with histopathological analyses, which showed typical cryptococcomas, as well as behavioral alterations. Altogether, this model permits evaluation of the ability of different *C. gattii* strains to affect the CNS, and also the host response to the fungus. The higher fungal burden in the lungs of mice infected with the L27/01 strain induced pro-inflammatory cytokines and, in turn, increased IL-10 concentrations, but this response did not prevent death. These data corroborate a previous study of murine cerebral infection with *C. gattii* [12]. In mice infected with the L27/01_F_ strain, higher cellular recruitment led to intense inflammatory reactions and alveolar thickening. TNF-α and IFN-γ increase the activity of phagocytes against cryptococcal cells; furthermore, IL-10 inhibits macrophage activation. Chemokines have a clear role in the recruitment of neutrophils to the lung, and mortality may result from an increased response of cytokine production [25], [26]. Overall, our data suggest that the immunomodulatory effects of L27/01_F_ may be linked to antigenic variation of surface proteins or carbohydrates [52] caused by fluconazole. In addition, the smaller size (and size distribution) of PS fibers compared to the parental strain may contribute to immunomodulation. However, we cannot exclude that molecular mechanisms such as epigenetic and chromosomal disomy [33], [35] may play a role in the phenotypic change of cryptococcal cells. Aneuploidy or isochromosome formation are also important in drug-resistant *C. albicans* [53], [54].

The altered behaviors of mice in the SHIRPA [27], [28] assay were similar to those described in humans with cryptococcosis [2]. The clinical domains not altered by the L27/01_F_ strain confirmed no dissemination to the CNS. To our knowledge, this is the first study to demonstrate behavioral changes in a murine model of cryptococcosis. The hippocampal neurodegeneration observed in mice infected with the L27/01 strain may account for the distinct behavioral profile [28], [55].

In conclusion, fluconazole causes changes in the PS capsule as an adaptation to the cellular stress caused by this antifungal agent. This phenotype persists, even upon removal of stress after many generations, and is correlated with *in vivo* susceptibility, revealing distinct profiles in recruitment of host cells and clinical manifestations of cryptococcosis, which has clinical implications. In addition, our results demonstrate a murine model suitable for pathogenicity studies and assessment of neurocryptococcosis and confirm that intracellular proliferation plays a key role in infection. We suggest that further studies with antifungal-resistant *C. gattii* strains are needed in order to better define the pathogenesis of infection and the mechanisms of fungal survival.

## Supporting Information

Table S1 **Primer sequences for real-time PCR.**
(DOC)

Table S2 **SHIRPA (evaluated parameters).**
(DOC)
