# Supplementary material for: Fluconazole Alters the Polysaccharide Capsule of Cryptococcus gattii and Leads to Distinct Behaviors in Murine Cryptococcosis
Source: PLoS One. 2014 Nov 13;9(11):e112669. doi: 10.1371/journal.pone.0112669 (PMC4231059; doi:10.1371/journal.pone.0112669)
Supplement: Table S1 — Primer sequences for real-time PCR. (DOC) [file pone.0112669.s001.doc]

**Fluconazole alters the polysaccharide capsule of *Cryptococcus gattii* and leads to distinct behaviors in murine cryptococcosis**

Julliana Ribeiro Alves Santos^1^, Rodrigo Assunção Holanda^1^, Susana Frases^2,3^, Mayara Bravim^2^, Glauber de S. Araujo^2,3^, Patrícia Campi Santos^1^, Marliete Carvalho Costa^1^, Maira Juliana Andrade Ribeiro^1^, Gabriella Freitas Ferreira^1^, Ludmila Matos Baltazar^1^, Aline Silva Miranda^4^, Danilo Bretas Oliveira^1^, Carolina Maria Araújo Santos^1^, Alide Caroline Lima Fontes^1^, Ludmila Ferreira Gouveia^1^, Maria Aparecida Resende-Stoianoff^1^, Jonatas Santos Abrahão^1^, Antônio Lúcio Teixeira^4^, Tatiane Alves Paixão^5^, Danielle G. Souza^1^; and Daniel Assis Santos^1*^

^1^Departamento de Microbiologia, Instituto de Ciências Biológicas, Universidade Federal de Minas Gerais, Minas Gerais, Brazil, ^2^Laboratório de Ultraestrutura Celular Hertha Meyer, Instituto de Biofísica Carlos Chagas Filho, Universidade Federal do Rio de Janeiro, UFRJ, RJ, Brazil. ^3^Laboratório de Biotecnologia (LABIO), Instituto Nacional de Metrologia, Normalização e Qualidade Industrial (INMETRO), Rio de Janeiro, Brazil. ^4^Laboratório Interdisciplinar de Investigação Médica, Faculdade de Medicina, Universidade Federal de Minas Gerais, Belo Horizonte, MG, Brazil. ^5^Departamento de Patologia Geral, Instituto de Ciências Biológicas, Universidade Federal de Minas Gerais.

*Corresponding author: Daniel de Assis Santos, Departamento de Microbiologia, Instituto de Ciências Biológicas, Universidade Federal de Minas Gerais, Av. Antonio Carlos, 6627, Pampulha, Belo Horizonte, Minas Gerais, Brazil, 31270-901. E-mail: [das@ufmg.br](mailto:das@ufmg.br); Fax: +55 31 3409 2733.

**Supplementary Data**

**Table S1.** Primer sequences for real-time PCR

| **Gene** | **Forward primer sequence (5'-3')** | **Reverse primer sequence (5'-3')** |
| --- | --- | --- |
| *C. gattii β-actin* | CTCCCCTTAACCCCAAGCAG | GAACGGCCTGGATAGAGACG |
| *ARF-1* | TGGCTGATGCCATGATTGGT | CTTGGCAGCAAGTTCCACAC |
| *CAP 59* | CGTACCCGTGGTCTGTTCAA | TTGTCACGGAGTTCGTGGAG |
| *CAP 64* | ACAACATGCTGGCACTGAGA | CTGGGACGGTCAGAAAGCAT |
| *ERG 11* | TGGCGTCTCTCAAATGGACC | ATCCCTTTTCGTCGTGCCAT |
| *MDR1* | CGTCGACTCCGAGAAGGA | CTCGCGACCAACGCTTATTG |
| *UXS-1* | TCACCATCCTCGAGTTTGCC | GAGGGTTGCCTTCCTCCTTC |
